# A method for predicting protein complex in dynamic PPI networks

**DOI:** 10.1186/s12859-016-1101-y

**Published:** 2016-07-25

**Authors:** Yijia Zhang, Hongfei Lin, Zhihao Yang, Jian Wang, Yiwei Liu, Shengtian Sang

**Affiliations:** College of Computer Science and Technology, Dalian University of Technology Dalian, Liaoning, China

## Abstract

**Background:**

Accurate determination of protein complexes has become a key task of system biology for revealing cellular organization and function. Up to now, the protein complex prediction methods are mostly focused on static protein protein interaction (PPI) networks. However, cellular systems are highly dynamic and responsive to cues from the environment. The shift from static PPI networks to dynamic PPI networks is essential to accurately predict protein complex.

**Results:**

The gene expression data contains crucial dynamic information of proteins and PPIs, along with high-throughput experimental PPI data, are valuable for protein complex prediction. Firstly, we exploit gene expression data to calculate the active time point and the active probability of each protein and PPI. The dynamic active information is integrated into high-throughput PPI data to construct dynamic PPI networks. Secondly, a novel method for predicting protein complexes from the dynamic PPI networks is proposed based on core-attachment structural feature. Our method can effectively exploit not only the dynamic active information but also the topology structure information based on the dynamic PPI networks.

**Conclusions:**

We construct four dynamic PPI networks, and accurately predict many well-characterized protein complexes. The experimental results show that (i) the dynamic active information significantly improves the performance of protein complex prediction; (ii) our method can effectively make good use of both the dynamic active information and the topology structure information of dynamic PPI networks to achieve state-of-the-art protein complex prediction capabilities.

**Electronic supplementary material:**

The online version of this article (doi:10.1186/s12859-016-1101-y) contains supplementary material, which is available to authorized users.

## Background

Prediction of protein complexes from protein-protein interaction (PPI) networks has become a key problem for revealing cellular function and organization of biological systems in post-genomic era. In a cell, proteins are central part of life activity. However, most of proteins are functional only after they are assembled into a protein complex which carry out almost all of the biochemical, signaling and functional processes in a cell. Protein complexes are of great importance for understanding the principles of cellular organization and function [1–3].

With the development of high-throughput techniques, such as yeast two-hybrid and mass spectrometry, a large amount of PPI data has been generated [4, 5]. As a result, large-scale PPI networks have been constructed for a wide range of organisms. Over the past decade, great efforts have been made to detect protein complexes in these PPI networks through the computational methods [6–13]. Most studies on protein complexes prediction have been focused on the static PPI networks [6]. Bader and Hogue [7] propose the Molecular Complex Detection (MCODE) algorithm that is one of the first computational methods to predict protein complexes. Markov Clustering (MCL) [8] can be applied to predict protein complexes by simulating random walks in PPI networks. Liu et al. [9] present a method called CMC (Clustering-based on Maximal Cliques) which identifies protein complexes based on maximal cliques. Chen et al. [10] propose a novel method using cliques as seeds and graph entropy to detect protein complexes. Wu et al. [11] present COACH algorithm to identify protein complexes based on the core-attachment structural feature. Since some proteins may belong to more than one protein complex, Nepusz et al. [12] propose ClusterONE algorithm to detect the overlapping protein complexes in a large PPI networks. In the past few years, some studies have integrated more biomedical resources, such as gene ontology (GO) and gene expression data, to improve the performance of protein complexes prediction. For example, Zhang et al. [13] integrate GO with PPI data to construct the ontology attributed PPI networks, and propose CSO algorithm to predict protein complexes in large ontology attributed PPI networks.

However, these methods described above only focus on the static PPI networks. In reality, the PPI network in a cell is not static but dynamic, which is changing over time, environments and different stages of cell cycles [14]. Generally, modeling biology systems as static PPI networks is a simple and efficient way to model biology systems. But static PPI networks loses all the temporal information which is critical to the understanding of the interaction between proteins in a cell. Therefore, the shift from static PPI networks to dynamic PPI networks is essential to predict protein complex accurately.

There are mainly two ways to construct dynamic PPI networks based on gene expression data and high-throughput PPI data. One major methodology to construct dynamic PPI networks is based on gene expression variance of each protein. In general, if a protein is at active time point, the expression level of the corresponding gene is at the peak point. Based on this assumption, Wang et al. [15] inject gene expression data into static PPI networks to construct dynamic PPI networks, and predict the protein complexes and the essential proteins. As an alternative, several studies have constructed dynamic PPI networks based on the differential co-expression correlations. For instance, Taylor et al. [16] observe multimodal distribution of correlation coefficients of gene expression using curated sources from the literatures. They analyze the human PPI networks and discover two types of hub proteins: intermodular hubs and intramodular hubs. Similarly, Lin et al. [17] reveal dynamic functional modules under conditions of dilated cardiomyopathy based on co-expression PPI networks.

Cellular systems are highly dynamic and responsive to cues from the environment [18, 19]. Both proteins and PPIs are changing over different stages of cell cycles. Therefore, not only the gene expression variance information but also the co-expression correlations information are necessary in the construction of an accurate dynamic PPI networks. In this study, we firstly integrate the two aspects to construct a dynamic PPI networks that can accurately model the dynamic processes in a cell. The active probability of both proteins and PPIs are calculated based on gene expression data and high-throughput PPI data. We then propose a clustering algorithm to predict the protein complexes in dynamic PPI networks. Finally, our method is compared with the state-of-the-art methods used for protein complex prediction. The advantages of the method, potential applications and improvements are discussed.

## Methods

### Construction of dynamic PPI networks

The gene expression data is very valuable to reveal the dynamic properties of proteins and PPIs. We integrate gene expression data with high-throughput PPI data to construct dynamic PPI networks. Based on gene expression data, we use both gene expression variance information and co-expression correlations information to calculate the active time point and active probability of each PPI in dynamic PPI networks.

Since the gene expression level of a protein will decrease after the protein has completed its function, different peak time points of gene expression value may represent the dynamic changes of protien activities. In general, a protein is active at the time point, when its related gene expression value is at the high level. A simple idea is to use a single global threshold for identifying the active time point of each protein. If the gene expression value of a protein is higher than the global threshold at a time point, the protein is active in the time point. Actually, it is very difficult to use a global threshold to identify the active time point of proteins. There are at least two reasons. On the one hand, the expression level of different protein in activity period is different. On the other hand, there is inevitable background noise in gene expression data. To solve these problems, Wang *et al.* [15] propose a three-sigma method to identify active time points of each protein in a cellular cycle. However, the active proteins with low expression values are likely to be filtered out even though using an active threshold for each gene. In this study, we calculate the active probability of each protein at different time points based on three-sigma method. We use equations (1) to calculate the *k*-sigma (k = 1,2,3) threshold for each gene expression data *p*.

1 $$ Thres{h}_k(p)=\alpha (p)+k\cdot \sigma (p)\cdot \left(1-\frac{1}{1+{\sigma}^2(p)}\right) $$

where *α*(*p*) and *σ*(*p*) are the arithmetic mean and the standard deviation (SD) of the gene expression data *p*, respectively. *Thresh*_*k*_ is determined by the values of *α*(*p*), *σ*^2^(*p*) and *k* (the times of sigma). Let *X* be a real random variable of normal distribution N(*α, σ*^*2*^). For any *k > 0*, P{|*X-α*| < *kσ*} = 2Φ(*k*)-1, where Φ(*.*) is the distribution function of the standard normal law. In particular, for *k* = 1,2,3 it follows that P{|*X-α*| < *σ*} = P{*α*-*σ* < *X < α* + *σ*} *≈* 0.6827, P{|*X-α*| < 2*σ*} *≈* 0.9545 and P{|*X-α*| < 3*σ*} *≈* 0.9973. Similarly, In the equation (1), the larger *k* is, the higher *Thresh*_*k*_ gets. A higher value of *Thresh*_*k*_ indicates that using more strict rules to identify the active time point of a protein. Let *G*_*i*_*(p)* be the gene expression value of the gene *p* at the time point *i*. For instance, based on the three-sigma rules, when *G*_*i*_*(p)*> *α*(*p*) + 3 ⋅ *σ*(*p*), the probability that the protein *p* (product of gene *p*) is active at the *i* time point is 99.7 %. But when *G*_*i*_*(p)*> *α*(*p*) + *σ*(*p*), the probability that the protein *p* (product of gene *p*) is active at the *i* time point is only 68.3 %.

We use a column Pr_i_ to represent the active probability of proteins at the time point *i*. Based on the above empirical rules, the active probability Pr_i_(*p*) of protein *p* at the time point *i* can be calculated as follows:

2 $$ { \Pr}_i(p)=\left\{\begin{array}{cc}\hfill 0.99\hfill & \hfill if\ {G}_i(p)\ge Thres{h}_3(p)\hfill \\ {}\hfill \begin{array}{c}\hfill 0.95\hfill \\ {}\hfill 0.68\hfill \\ {}\hfill 0\hfill \end{array}\hfill & \hfill \begin{array}{c}\hfill if\  Thres{h}_3(p)>{G}_i(p)\ge Thres{h}_2(p)\hfill \\ {}\hfill if\  Thres{h}_2(p)>{G}_i(p)\ge Thres{h}_1(p)\hfill \\ {}\hfill if\ {G}_i(p)< Thres{h}_1(p)\hfill \end{array}\hfill \end{array}\right. $$

Thus, we can use four levels (0.99, 0.95, 0.68 and 0) to represent the active probability of each protein at the time point *i*.If the value of *G*_*i*_*(p)* is lower than *Thresh*_*k*_*(p)*, the active probability is 0. It indicates that the protein p is not active in the *i* time point, when Pr_i_(*p*) is equal to 0. This method not only identifies the active time points for each protein, but also distinguishes the active level of the protein by its active probability, which is more reasonable than both global threshold methods and active threshold methods. But we also note that, in some extreme cases, our method still cannot accurately identify the active time points of proteins. The whole activity PPI networks *Act*_*i*_ are built for each time point:

3 $$ Ac{t}_i={ \Pr}_i{{ \Pr}_i}^T $$

where Pr_i_ is a column vector representing the activity of all proteins at time i and Pr_i_^T^ is the transpose of the column vector Pr_i_.

Coexpression correlation coefficient is used as a measure of coexpressed genes having the same expression variance patterns across different conditions, which is a strong indicator of protein functional associations. Zhang et al. use the Pearson correlation coefficient (normalized to the range of 0 to 1) to calculate the coexpression correlation of gene expression data and build coexpression protein networks at different time points [20]. We use the same method to calculate the coexpression protein networks *Coe. Coe*_*i*_ denotes the coexpression protein networks *Coe* at the time point *i*. Calculation of correlation coefficient requires multiple sequential expression data that cover a period of time. We set a time window on the original expression data, which covers three sequential time points. When *i* is the current time point, the time window covers three time points including *i-1*, *i* and *i + 1*. We use a predefined threshold to filter the small value of correlation coefficient in *Coe*_*i*_ as follows:

4 $$ Co{e}_i\left(m,n\right)=\left\{\begin{array}{cc}\hfill \left|P\_ Correlatio{n}_i\left(m,n\right)\right|\hfill & \hfill \left|P\_ Correlatio{n}_i\left(m,n\right)\right|\ge Pre\_ thresh\hfill \\ {}\hfill 0\hfill & \hfill \left|P\_ Correlatio{n}_i\left(m,n\right)\right|<Pre\_ thresh\hfill \end{array}\right. $$

where *P_Correlation(m,n)* is the Pearson correlation coefficient between the protein *m* and protein *n* at the time point *i. Pre_thresh* is the predefined threshold, and we can choose the optimal value for *Pre_thresh* by preliminary experiments. In our experiments, we set *Pre_thresh* as 0.5.

The high-throughput PPI data can construct a static PPI networks. Let *adj_SPN* denote the static PPI networks adjacency matrix. Integrating *Act*, *Coe* and *adj_SPN*, we can calculate the dynamic PPI networks adjacency matrix *adj_DPN* at the time point *i* as follows:

5 $$ adj\_DP{N}_i=Ac{t}_i\circ Co{e}_i\circ adj\_SPN $$

where ○ represents element-wise multiplication. Equation (5) integrates the topology information of static PPI networks with the dynamic information of gene expression effectively. In respect of dynamic information, Equation (5) takes into account the active probability of each proteins as well as the coexpression correlation of each PPI. Based on equation (5), we can calculate an active probability for each PPI in the dynamic PPI networks at different time points. The value of active probability of each PPI takes ranges of 0 to 1.

**Fig. 1:**
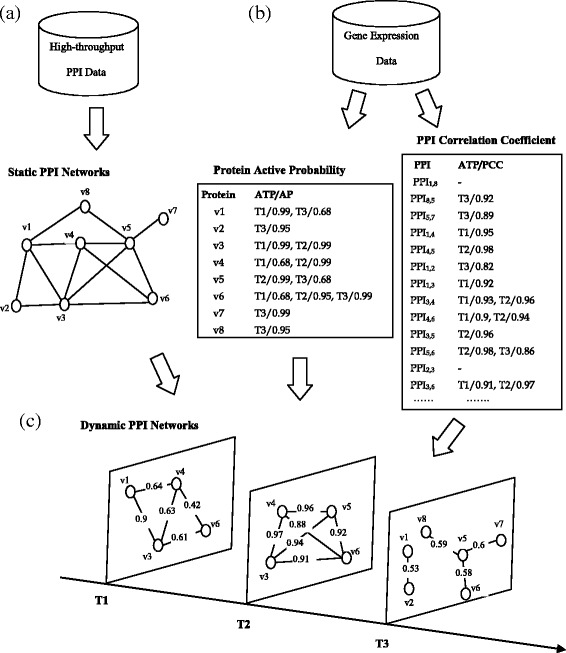
An illustration example of dynamic PPI networks construction. **a** construction of static PPI networks based on high-throughput PPI data. **b** calculation of dynamic information based on gene expression data. ATP, AP and PCC denote active time points, active probability and Pearson correlation coefficient, respectively. **c** construction of dynamic PPI networks

Figure 1 shows an illustration example of the dynamic PPI networks construction. In Fig. 1(a), we construct static PPI networks based on high-throughput PPI data, which don’t contain any temporal or dynamic information. In Fig. 1(b), we exploit gene expression data to calculate the active probability of proteins and the Pearson correlation coefficient of PPIs, respectively. It can be seen that each protein in the static PPI networks is associated with the active time points and the active probability. Based on the equation (2), the active probability of proteins only include three values 0.99, 0.95 and 0.68. For instance, the protein *v*_*1*_ has two active time points (*T1* and *T3*), and its active probability is 0.99 at *T1* active time point. A PPI in the static PPI networks (Fig. 1a) is active at the time point *i,* if the two proteins associated with this PPI are both active at the time point *i.* Then, we calculate the Pearson correlation coefficient between the two proteins at the active time point *i.* In Fig. 1(b), “PPI_1,8_” denotes the PPI between *v*_*1*_ and *v*_*8*_, and “-” denotes the Pearson correlation coefficient is lower than the predefined threshold. In Fig. 1(c), to construct dynamic PPI networks, we integrate the topology information (Fig. 1a) of static PPI networks with the dynamic information (Fig. 1b) calculated based on gene expression data. We use equation (5) to calculate the probability value of each PPI in the dynamic PPI networks (Fig. 1c).

### Protein complex prediction from dynamic PPI networks

Dynamic PPI networks can effectively represent not only the topology structure but also the dynamic information of PPI networks. A dynamic PPI network generally consists of a serial of active PPI subnetworks. For example, the dynamic PPI network in Fig. 1(c) consists of three active PPI subnetworks. Let *DPN* denote a dynamic PPI networks that includes *Tk* active PPI subnetworks {*DPN*_*T1*_, *DPN*_*T2*_, …, *DPN*_*Tk*_}. {*adj_DPN*_*T1*_, *adj_DPN*_*T2*_, …, *adj_DPN*_*Tk*_} is the adjacency matrices of the *DPN* at *T1,T2, …,Tk* active time points. Given a subgraph *SG* in an active PPI subnetworks *DPN*_*Ti*_, let *V*_*SG*_ and *E*_*SG*_ denote the set of proteins and PPIs in *SG*, respectively. The density of *SG* is defined as follows:

6 $$ Densit{y}_{Ti}(SG)=\frac{2\times {\displaystyle {\sum}_{e\left(u,v\right)\in {E}_{SG}}adj\_DP{N}_{Ti}\left(u,v\right)}}{\left|{V}_{SG}\right|\times \left(\left|{V}_{SG}\right|-1\right)} $$

Given a subgraph *SG* in the active PPI subnetworks *DPN*_*Ti*_, a protein *v* in the active PPI subnetworks *DPN*_*Ti*_, and *v*∉*V*_*SG*_, the attached score between *v* and *V*_*SG*_ in the *DPN*_*Ti*_, is given as:

7 $$ Attach\_ Scor{e}_{Ti}\left(v,{V}_{SG}\right)=\frac{{\displaystyle {\sum}_{u\in {V}_{SG}}adj\_DP{N}_{Ti}\left(u,v\right)}}{\left|{V}_{SG}\right|} $$

The edges in the active PPI subnetworks *DPN*_*Ti*_ contribute differently for protein complex prediction. The cluster score of edge *e(u,v)* in *DPN*_*Ti*_ is defined as follows:

8 $$ Cluster\_ Scor{e}_{Ti}\left(e\left(u,v\right)\right)=adj\_DP{N}_{Ti}\left(u,v\right)\times \frac{2\times \left(\left|{N}_u\cap {N}_v\right|+1\right)}{\left|{N}_u\right|+\left|{N}_v\right|} $$

where *N*_*u*_ and *N*_*v*_ denote the neighbors of protien *u* and protein *v* respectively. |*N*_*u*_∩*N*_*v*_ | denotes the common neighbors of *u* and *v*. In the respect of topology structure of an active PPI subnetworks, the more common neighbors *u* and *v* share, the closer the interaction of two proteins *u* and *v* is. Cluster score can effectively balance the effect between the topology closeness and the active probability of each PPI in the active PPI subnetworks.

A protein complex is a group of proteins assembled by multiple PPIs at the same time and place [1, 21]. Moreover, some analysis of protein complexes has revealed their core-attachment organization feature [22, 23]. Our method for predicting protein complexes from a whole dynamic PPI networks involves two phases. In the first phase, our method predicts candidate protein complexes from all active PPI subnetworks in turn. All candidate protein complexes are added into *Candidate_complex*. In the second phase, our method filters the candidate complexes set *Candidate_complex* to remove the highly overlapped protein complexes.

The description of subroutine for detecting possible protein-complex cores is shown in Algorithm 1. Firstly our method calculates the *Cluster_Score* of all edges in *DPN*_*Ti*_ based on equation (8). The edge will be added into *Seed_set*, if its *Cluster_Score* is not less than *Complex_thresh* that is a predefined threshold parameter. The effect of *Complex_thresh* will be discussed in our experiments. Secondly, we rank all seed edges of *Seed_set* in descending order of their *Cluster_Score* value, denote as *Seed_list =* (*S*_*1*_, *S*_*2*_*,…, S*_*n*_). The top ranked seed edge *S*_*1*_ was then inserted into the *Temp_Candidate_core* set *CORE* and removed from *Seed_list.* We augment the seed edge *S*_*1*_ to generate the core structure by adding the suitable neighbor proteins one by one at line 13-17. If the *Density* value of the core structure is not less than *Complex_thresh* when adding the neighbor protein *p*, it will be added into the core structure. To ensure that the core structure are non-overlapping, the overlapped seed edges are removed from *Seed_list* at line 19-21. Finally, the attachment proteins are detected for each core structure based on the *Attach_Score* that is calculated by equation (7). The attachment proteins are added into the core structure to form the candidate protein complex.

The candidate protein complexes in *Candidate_complex* generally overlap with each other. The description of subroutine for postprocessing of overlapped protein complexes is shown in Algorithm 2. All candidate protein complexes are ranked in descending order of their *Density* value (*Candidate_list =* (*cc*_*1*_, *cc*_*2*_*,…, cc*_*n*_)) at line 2-5. The candidate protein complex associated with highest *Density* value in *Candidate_list* is added into *Complex_set* and removed from *Candidate_list*. For any other candidate protein complex *cc*_*i*_ ∈*Candidate_list*, we check the overlapped degree between *cc*_*i*_ and *cc*_*1*_. If the overlapped degree is larger than the *Overlap_thresh* that is a predefined threshold parameter, *cc*_*i*_ is directly removed from *Candidate_list* at line 9-12*.* After preliminary experiments, the *Overlap_thresh* is set as 2/3 in our experiments. These steps are repeated until *Candidate_list* is empty. Consequently, the final protein complex set *Complex_set* is generated.

## Results and Discussion

In this section, the datasets and evaluation metrics used in the experiments are described. The impact of the *Complex_thresh* parameter is assessed and discussed. Then, our method is compared with current state-of-the-art protein complex prediction methods. Finally, we present an example of predicted protein complex to illustrate the advantages of our method. This implement of our algorithm and the experimental datasets are avialable in the Additional files 1, 2 and 3.

### Datasets and evaluation metrics

In our experiments, we choose four high-throughput yeast PPI datasets including Gavin dataset [23], Krogan dataset [24], MIPS dataset [25] and STRING dataset [26], respectively. In particular, STRING dataset is now one of the largest PPI datasets, which integrates yeast PPI data from the four sources, including high-throughput data, co-expression data, genomic context data and biomedical literature data. The statistics of the four yeast PPI datasets is listed in Table 1.

**Table 1:** The statistics of PPI datasets in experiments

High-throughput PPI data	Proteins	Interactions
Gavin dataset	1430	6531
Krogan dataset	2675	7080
MIPS dataset	3950	11119
STRING dataset	5970	99786

The gene expression data used in our experiment is GSE3431 [27] downloaded from Gene Expression Omnibus (GEO). GSE3431 gene expression data is an expression profiling of yeast by array affymetrix, which includes the expression profiles of 9,335 probes. The experimental design of GSE3431 is 12 time intervals per cycle, and approximately 25 min per time interval. Therefore, there are 12 active time points (*T1,T2,…,T12*) for each gene in a cycle. We construct four dynamic PPI networks to integrate high-throughput PPI data and gene expression data. DPN_Gavin, DPN_Krogan, DPN_MIPS and DPN_STRING are constructed by integrating gene expression data GSE3431 with the Gavin dataset, Krogan dataset, MIPS dataset and STRING dataset, respectively.

The benchmark protein complex dataset CYC2008 [28] includes 408 manually curated heterometric protein complexes, which is used to evaluate the protein complexes predicted by our method.

To assess the quality of predicted protein complexes, we match generated complexes with the benchmark complex set CYC2008. Let *P*(*V*_*P*_*, E*_*P*_) be a predicted complex and *B*(*V*_*B*_*, E*_*B*_)) be a known complex. We define the neighborhood affinity score *NA(P,B)* between *P*(*V*_*P*_*, E*_*P*_)and *B*(*V*_*B*_*, E*_*B*_)) as follows:

9 $$ NA\left(P,B\right)=\frac{{\left|VP\cap VB\right|}^2}{\left|VP\right|\times \left|VB\right|} $$

If *NA(P,B)* is 1, it means that the identified complex *P(V*_*P*_*, E*_*P*_*)* has the same proteins as a known complex *B(V*_*B*_*, E*_*B*_*)*. On the contrary, if *NA(P,B)* is 0, it indicates no shared protein between *P(V*_*P*_*, E*_*P*_*)* and *B(V*_*B*_*, E*_*B*_*)*. We considered *P(V*_*P*_*, E*_*P*_*)* and *B(V*_*B*_*, E*_*B*_*)* to match each other if *NA(P,B)* was larger than 0.2, which is the same as most methods for protein complex identification [6].

Precision, recall and *F-score* have been used to evaluate the performance in most of previous complex prediction studies, which are defined as follows:

10 $$ precision=\frac{Nci}{\left| Identified\_ Set\right|} $$

11 $$ recall=\frac{Ncb}{\left| Benchmark\_ Set\right|} $$

12 $$ F\hbox{-} score=\frac{2 precision\cdot recall}{\left( precision+ recall\right)} $$

where *N*_*ci*_ is the number of identified complexes which match at least one known complex, and *N*_*cb*_ is the number of known complexes that match at least one identified complex. *Identified_Set* denotes the set of complexes identified by a method and *Benchmark_Set* denotes the gold standard dataset. Precision measures the fidelity of the predicted protein complex set. Recall quantifies the extent to which a predicted complex set captures the known complexes in the benchmark set. *F-score* provides a reasonable combination of both precision and recall, and can be used to evaluate the overall performance. To keep our evaluation metrics as the same as the most studies, we choose *F*-score as the major evaluation metrics.

Recently, sensitivity (Sn), positive predictive value (PPV) and accuracy (Acc) have also been used to evaluate protein complex prediction tools. Acc represents a tradeoff between Sn and PPV. The advantage of the geometric mean is that it yields a low score when either Sn or PPV are low. A high degree of accuracy thus requires a high performance for both criteria. These definitions have been described in detail by Li *et al.* [6]. In our experiments, we also report Sn, PPV and Acc of our method on different PPI datasets.

### The effect of threshold parameters

In this experiment, we evaluate the effect of the threshold parameter *Complex_thresh* for protein complex prediction task on different dynamic PPI networks. As described in Algorithm 1, the *Complex_thresh* determines the number of seed edges in the *Seed_set*, as well as the *Density* value of the core structure. The range of *Complex_thresh* is from 0 to 1. We can choose the optimal value of *Complex_thresh* by the experimental approach.

We first evaluate the effect of *Complex_thresh* on DPN_Gavin. The detailed experimental results on DPN_Gavin with different *Complex_thresh* are shown in Table 2. The highest value in each row is in bold. As shown in Table 2, the number of predicted protein complexes continues to decrease as the value of *Complex_thresh* takes from 0 to 1. When *Complex_thresh* = 0, our method can predict 623 protein complexes on the DPN_Gavin. On the contrary, our method cannot predict any protein complexes on the same DPN_Gavin when *Complex_thresh* =1.0. Based on the equation (8), the Cluster Score is smaller than 1 in theory. In other words, it is impossible to generate any seed edge in *Seed_set* if we set *Complex_thresh* =1.0. Overall, with the increase of *Complex_thresh,* the recall, Sn and Acc are decreased in Table 2. The precision achieves the highest value of 0.784 when *Complex_thresh* =0.7, and the PPV achieves the highest value of 0.906 when *Complex_thresh* =0.9. The major metrics *F*-score is ranged from 0.048 to 0.524. When *Complex_thresh* =0.1, the *F*-score achieves the highest value of 0.524.

**Table 2:** The effect of Complex_thresh on protein complex prediction performance on DPN_Gavin

*Complex_thresh*	#Complexes	P	R	F	Sn	PPV	Acc
0	623	0.549	**0.468**	0.505	**0.43**	0.619	**0.516**
0.1	447	0.662	0.434	**0.524**	0.413	0.617	0.505
0.2	325	0.695	0.385	0.495	0.379	0.624	0.486
0.3	238	0.752	0.304	0.433	0.31	0.638	0.445
0.4	181	0.74	0.25	0.374	0.24	0.653	0.395
0.5	130	0.708	0.174	0.279	0.178	0.687	0.349
0.6	87	0.724	0.12	0.206	0.118	0.72	0.291
0.7	51	**0.784**	0.088	0.159	0.074	0.71	0.23
0.8	30	0.733	0.059	0.109	0.049	0.67	0.181
0.9	13	0.769	0.025	0.048	0.016	**0.906**	0.119
1	0	-	-	-	-	**-**	-

F: *F-score*, P: precision, R: recall. The highest score of each row is shown in bold

Then, we evaluate the effect of *Complex_thresh* on the DPN_Krogan and DPN_MIPS. The detailed experimental results with different *Complex_thres* are shown in Tables 3 and 4. The experimental results of *Complex_thresh* on the DPN_Krogan and DPN_MIPS are similar to the experimental results on the DPN_Gavin. When *Complex_thresh* =0.1, our method achieves the highest *F*-score of 0.52 and 0.372 on the DPN_Krogan and DPN_MIPS, respectively. Based on these experimental results on three DPNs, it can be seen that our method can achieve high performance for protein complex prediction by setting *Complex_thresh* = 0.1.

**Table 3:** The effect of Complex_thresh on protein complex prediction performance on DPN_Krogan

*Complex_thresh*	#Complexes	P	R	F	Sn	PPV	Acc
0	1246	0.388	**0.691**	0.497	**0.488**	0.673	**0.573**
0.1	816	0.464	0.591	**0.52**	0.448	0.677	0.551
0.2	546	0.526	0.512	0.519	0.401	0.685	0.524
0.3	353	0.598	0.363	0.451	0.316	0.685	0.465
0.4	223	0.619	0.255	0.361	0.217	0.705	0.391
0.5	144	0.632	0.181	0.282	0.149	0.721	0.328
0.6	97	0.608	0.115	0.194	0.101	0.777	0.279
0.7	52	**0.673**	0.071	0.129	0.061	0.78	0.219
0.8	37	0.595	0.059	0.107	0.04	0.781	0.177
0.9	15	0.533	0.025	0.047	0.015	**0.857**	0.112
1	0	-	-	-	-	**-**	-

F: *F-score*, P: precision, R: recall. The highest score of each row is shown in bold

**Table 4:** The effect of Complex_thresh on protein complex prediction performance on DPN_MIPS

*Complex_thresh*	#Complexes	P	R	F	Sn	PPV	Acc
0	1895	0.239	**0.681**	0.353	**0.413**	0.608	**0.501**
0.1	1145	0.274	0.576	**0.372**	0.382	0.61	0.483
0.2	611	0.327	0.404	0.361	0.313	0.634	0.446
0.3	321	0.364	0.26	0.303	0.224	0.644	0.38
0.4	192	0.396	0.174	0.242	0.141	0.633	0.299
0.5	101	0.426	0.11	0.175	0.089	0.642	0.239
0.6	57	**0.439**	0.061	0.108	0.046	0.726	0.182
0.7	23	0.348	0.02	0.037	0.016	0.73	0.109
0.8	13	0.231	0.005	0.01	0.003	**1**	0.05
0.9	11	0.182	0.005	0.01	0.003	**1**	0.06
1	0	-	-	-	-	**-**	-

F: *F-score*, P: precision, R: recall. The highest score of each row is shown in bold

### Comparison with other methods

In this experiment, we compare our method with the following established leading protein complex prediction methods: CSO [13], Cluster ONE [12], COACH [11], CMC [9], HUNTER [29], and MCODE [7] (Table 5). These methods are used to compare the performance in most of recent complex prediction studies. In this experiment, we set *Complex_thresh* =0.1. To equally compare the performance, we test all comparison methods on the Gavin, Krogan and MIPS dataset, respectively, and choose the optimal parameters. The highest value in each row was shown in bold.

**Table 5:** Performance comparison with other protein complex prediction methods

PPI data	Methods	#Complexes	P	R	F	Sn	PPV	Acc
Gavin data	Our method	447	0.662	**0.434**	**0.524**	0.413	**0.617**	0.505
	CSO	174	0.645	0.302	0.411	**0.476**	0.534	0.503
	Cluster ONE	243	0.502	0.324	0.393	0.46	0.597	**0.524**
	COACH	326	0.525	0.331	0.406	0.44	0.547	0.49
	CMC	120	0.608	0.218	0.321	0.371	0.606	0.474
	HUNTER	69	**0.87**	0.206	0.333	0.386	0.508	0.443
	MCODE	66	0.727	0.142	0.238	0.277	0.513	0.377
Krogan data	Our method	816	0.464	**0.591**	**0.52**	**0.448**	0.677	**0.551**
	CSO	190	0.726	0.331	0.455	0.411	0.642	0.514
	Cluster ONE	240	0.579	0.328	0.419	0.398	**0.681**	0.521
	COACH	345	0.617	0.343	0.441	0.432	0.544	0.485
	CMC	111	0.748	0.235	0.358	0.381	0.589	0.474
	HUNTER	74	**0.865**	0.199	0.323	0.374	0.569	0.462
	MCODE	76	0.724	0.157	0.258	0.255	0.583	0.385
MIPS data	Our method	1145	0.274	**0.576**	**0.372**	0.382	0.61	**0.483**
	CSO	192	0.495	0.289	0.365	0.286	0.568	0.403
	Cluster ONE	256	0.359	0.23	0.281	0.243	**0.668**	0.403
	COACH	448	0.301	0.289	0.295	0.336	0.311	0.323
	CMC	168	0.429	0.211	0.283	**0.389**	0.318	0.352
	HUNTER	52	**0.654**	0.11	0.189	0.296	0.286	0.291
	MCODE	85	0.447	0.115	0.183	0.19	0.503	0.309
STRING data	Our method	1240	0.324	**0.586**	**0.417**	0.836	0.404	0.581
	Cluster ONE	893	0.151	0.245	0.187	0.846	**0.459**	**0.623**
	COACH	1645	0.186	0.292	0.227	**0.955**	0.12	0.338
	HUNTER	5	**0.5**	0.01	0.019	0.104	0.298	0.176
	MCODE	393	0.092	0.09	0.091	0.675	0.242	0.405

#Complexes refers to the number of predicted complexes. F: *F-score*, P: precision, R: recall. The highest score of each approach is shown in bold

Firstly, we compare these methods on the Gavin dataset. As shown in Table 5, our method achieves the highest *F*-score of 0.524, recall of 0.434 and PPV of 0.617, respectively, which significantly outperforms other methods. CSO achieves a high *F*-score of 0.411 and the highest Sn of 0.476, which exploits the GO annotation data to improve the performance of protein complexes identification. HUNTER achieves the highest precision of 0.87. But the recall of HUNTER is only 0.206, which leads to a low *F*-score of 0.333. Cluster ONE achieve the highest Acc of 0.524. We also note that our method can predict more protein complexes than other methods. For example, our method can predict 447 protein complexes on the Gavin dataset. In contrast, MCODE and HUNTER only identify 66 and 69 protein complexes on the Gavin dataset, respectively.

Secondly, we compare these methods on the Krogan dataset and MIPS dataset. On the Krogan dataset, it can be seen that the results on the Krogan dataset are similar to the results on the Gavin dataset. On the Krogan datasets, our method achieves the highest *F*-score of 0.52, recall of 0.591, Sn of 0.448 and Acc of 0.551. CSO also achieves a high *F*-score of 0.455, which is only inferior to our method. HUNTER and Cluster ONE achieve the highest precision of 0.865 and PPV of 0.681, respectively. On the MIPS datasets, our method also achieves the highest *F*-score of 0.372, recall of 0.576 and Acc of 0.483, respectively. HUNTER, CMC and Cluster ONE achieve the highest precision of 0.654, Sn of 0.389 and PPV of 0.668, respectively.

Thirdly, we compared these methods on the STRING dataset. STRING dataset is much larger than other three PPI datasets, which contains 99786 PPIs. Due to the complexity of the PPI network constructed by STRING dataset, it is much more difficult to predict protein complex on STRING dataset than other three datasets. From Table 5, it can be seen that the major metrics *F*-score of all comparison methods except for our method on STRING dataset are clearly inferior to the *F*-score on other three datasets. The compared experiments were conducted on a 3.3GHz four-Core Intel I5 CPU and 8GB main memory. Actually, CSO and CMC methods cannot output the results on STRING dataset, because the clique mining algorithms used by the two methods are very memory and CPU cycle consuming in such large PPI networks. Compared with other methods, our method firstly use STRING dataset and gene expression data to construct a whole dynamic PPI networks DPN_STRING which consists of 12 active PPI subnetworks, {*DPN*_*T1*_, *DPN*_*T2*_, *…, DPN*_*T12*_}. Then, our method predicts the protein complexes from these active PPI subnetworks in turn. Since each active PPI subnetwork is much smaller than the whole static PPI networks, our method is more suitable to deal with very large PPI dataset such as STRING than other methods. From Table 6, it can be seen that the computational time of our method is far less than other methods on STRING dataset. In particular, our method can also achieve the high *F*-score of 0.417 and recall of 0.586 on STRING dataset.

**Table 6:** Performance comparison in computational time

Methods	Gavin data	Krogan data	MIPS data	STRING data
Our method	1,624 ms	2,150 ms	3,487 ms	68,719 ms
CSO	173,562 ms	40,954 ms	215,476 ms	>12 h
Cluster ONE	2,166 ms	3,154 ms	4,317 ms	183,634 ms
COACH	1,783 ms	1,207 ms	3,772 ms	3,351,694 ms
CMC	339 ms	1,397 ms	1,450 ms	>12 h
HUNTER	172 ms	3,322 ms	5,451 ms	1,222,027 ms
MCODE	1,879 ms	1,985 ms	3,732 ms	785,616 ms

In summary, our method not only effectively integrates gene expression data and high-throughput PPI data to construct dynamic PPI networks, but also makes good use of dynamic information of dynamic PPI networks to improve the performance of protein complex prediction. Our method is competitive or superior to the current protein complexes identification methods, and achieves the state-of-the-art performance on different yeast PPI datasets.

### Examples of predicted complexes

**Fig. 2:**
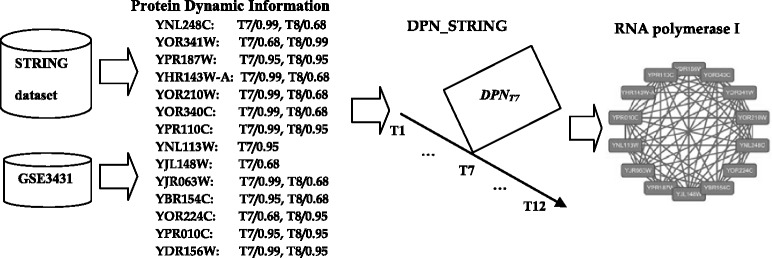
RNA polymerase I complex predicted by our method on STRING dataset

Figure 2 shows the RNA polymerase I complex predicted exactly by our method on STRING dataset. Based on the gene expression data and STRING dataset, our method firstly calculates the protein dynamic information, and then constructs the DPN_STRING. From Fig. 2, it can be seen that all proteins of RNA polymerase I share the common active time point *T*7. This indicates that all these proteins will be active in the active PPI subnetwork *DPN*_*T7*_. Eventually, our method exactly predicts the RNA polymerase I complex from the PPI subnetwork *DPN*_*T7*_ rather than from the whole PPI network. Furthermore, this result suggests that the life period of the RNA polymerase I is at *T7* time point. Compared with other methods, our method can predict the RNA polymerase I exactly from the very large PPI dataset STRING, as well as the active time point of the complex.

**Fig. 3:**
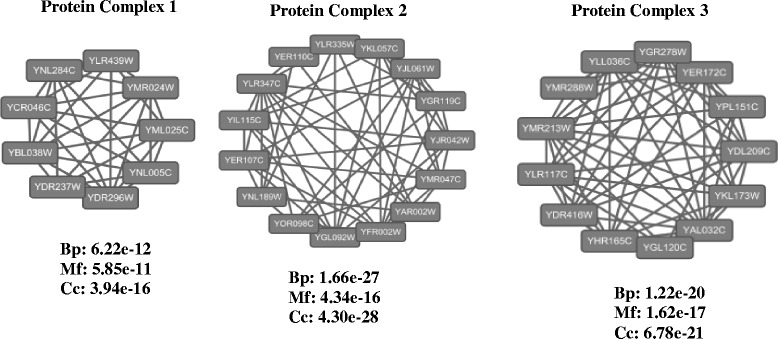
Examples of protein complexes predicted by our method

In Fig. 3, we present some examples of the predicted complexes which are not matched with the benchmark dataset. We evaluate the biological significance of these predicted complexes. In this experiment, we use SGD’s GO::TermFinder to calculate the p-value of each predicted complex, which is the statistical significance of the occurrence of an predicted complex with respect to GO data. In general, an predicted complex is considered to be statistically significant if the p-value is less than 0.01, and a smaller p-value generally represents higher biological meaning. From Fig. 3, it can be seen that the three ed complexes both have very low p-value and highly local density. Therefore, the results provide clues for biologists to verify and find new protein complexes.

## Conclusions

We integrate gene expression data and high-throughput PPI data to construct dynamic PPI networks. Based on gene expression data, we calculate the active time point and the active probability of each protein and PPI. Compared with static PPI networks, dynamic PPI networks can effectively represent both the dynamic active information and the topology structure information of PPI networks. Using dynamic PPI networks, we develop a novel method for protein complex prediction. Experimental comparisons on different PPI datasets show that our approach achieves the state-of-the-art PPI performance. In the future, we will cooperate with biomedical experts to further validate the protein complexes identified by our method. We will also attempt to apply our method to analysis other organisms.

## Additional files

Additional file 1: Source code for dynamic protein complexes identification. This implement of our algorithm runs under Windows OS. The main requirement is python 2.7 or later and numpy. (PY 19 kb) Additional file 2: Krogan PPI data. (TXT 117 kb) Additional file 3: GSE3431 gene expression data. (TXT 4397 kb)
